# Knowledge mapping of neonatal electroencephalogram: A bibliometric analysis (2004–2022)

**DOI:** 10.1002/brb3.3483

**Published:** 2024-04-28

**Authors:** Ruijie Zhang, Lifeng Shi, Lu Zhang, Xinao Lin, Yunlei Bao, Feng Jiang, Chuyan Wu, Jimei Wang

**Affiliations:** ^1^ Department of Neonatology Obstetrics and Gynecology Hospital of Fudan University Shanghai China; ^2^ Department of Rehabilitation Medicine The First Affiliated Hospital of Nanjing Medical University Nanjing China

**Keywords:** bibliometric analysis, CiteSpace (rrid: scr_025121), electroencephalogram, neonate, VOSviewer (rrid:scr_023516)

## Abstract

**Background:**

Electroencephalography (EEG), a widely used noninvasive neurophysiological diagnostic tool, has experienced substantial advancements from 2004 to 2022, particularly in neonatal applications. Utilizing a bibliometric methodology, this study delineates the knowledge structure and identifies emergent trends within neonatal EEG research.

**Methods:**

An exhaustive literature search was conducted on the Web of Science Core Collection (WoSCC) database to identify publications related to neonatal EEG from 2004 to 2022. Analytical tools such as VOSviewer, CiteSpace, and the R package “bibliometrix” were employed to facilitate this investigation.

**Results:**

The search yielded 2501 articles originating from 79 countries, with the United States and England being the predominant contributors. A yearly upward trend in publications concerning neonatal EEG was observed. Notable research institutions leading this field include the University of Helsinki, University College London, and University College Cork. *Clinical Neurophysiology* is identified as the foremost journal in this realm, with *Pediatrics* as the most frequently co‐cited journal. The collective body of work from 9977 authors highlights Sampsa Vanhatalo as the most prolific contributor, while Mark Steven Scher is recognized as the most frequently co‐cited author. Key terms such as “seizures,” “epilepsy,” “hypoxic‐ischemic encephalopathy,” “amplitude‐integrated EEG,” and “brain injury” represent the focal research themes.

**Conclusion:**

This bibliometric analysis offers the first comprehensive review, encapsulating research trends and progress in neonatal EEG. It reveals current research frontiers and crucial directions, providing an essential resource for researchers engaged in neonatal neuroscience.

## 1 INTRODUCTION

Electroencephalography (EEG) is a methodological approach that records the electrical activity generated by the brain's neurons, captured from the scalp's surface (Biasiucci et al., 2019). Video‐electroencephalography (vEEG) is acknowledged as the gold standard for continuous neurophysiological monitoring in neonates (Shellhaas et al., 2011). Despite its effectiveness, the implementation of continuous vEEG in neonatal intensive care units (NICUs) encounters substantial challenges (Boylan & Stevenson et al., 2013; Boylan et al., 2010; Dilena et al., 2019; Hellström‐Westas, 2018). These challenges encompass the scarcity of necessary equipment, a shortage of trained personnel, and a lack of expertise required for long‐term continuous vEEG monitoring. As a result, amplitude‐integrated EEG (aEEG), previously known as cerebral function monitor (CFM), has been adopted as a simplified alternative for continuous brain monitoring, utilizing one to three channels and noted for its practicality (Boylan & Stevenson et al., 2013).

EEG, as a noninvasive diagnostic tool, enables continuous monitoring of cerebral cortex functionality. It is extensively applied in clinical settings, particularly for assessing epileptic seizures, sleep patterns, and functional neurological outcomes (Castro Conde et al., 2018; del Rio‐Bermudez & Blumberg, 2018). Additionally, EEG is pivotal in detecting changes in brain maturation across developmental stages, from infancy to adolescence, and serves as a predictive marker for neural developmental anomalies in premature infants (Stevenson et al., 2020; Vandenbosch et al., 2019).

Bibliometrics emerges as a contemporary methodology for analyzing research output metrics, offering a dual qualitative and quantitative insight into the literature by evaluating parameters such as country, journal, organization, and keywords (Muslu, 2018). This approach aids in developing guidelines, pinpointing research focal points, and forecasting future research directions (Guler et al., 2016). To date, no bibliometric analysis has been conducted on neonatal EEG. This study aims to utilize bibliometric analysis software to identify key research areas in neonatal EEG from 2004 to 2022 and delineate prospective research pathways.

## 2 MATERIALS AND METHODS

### 2.1 Data source

**FIGURE 1:**
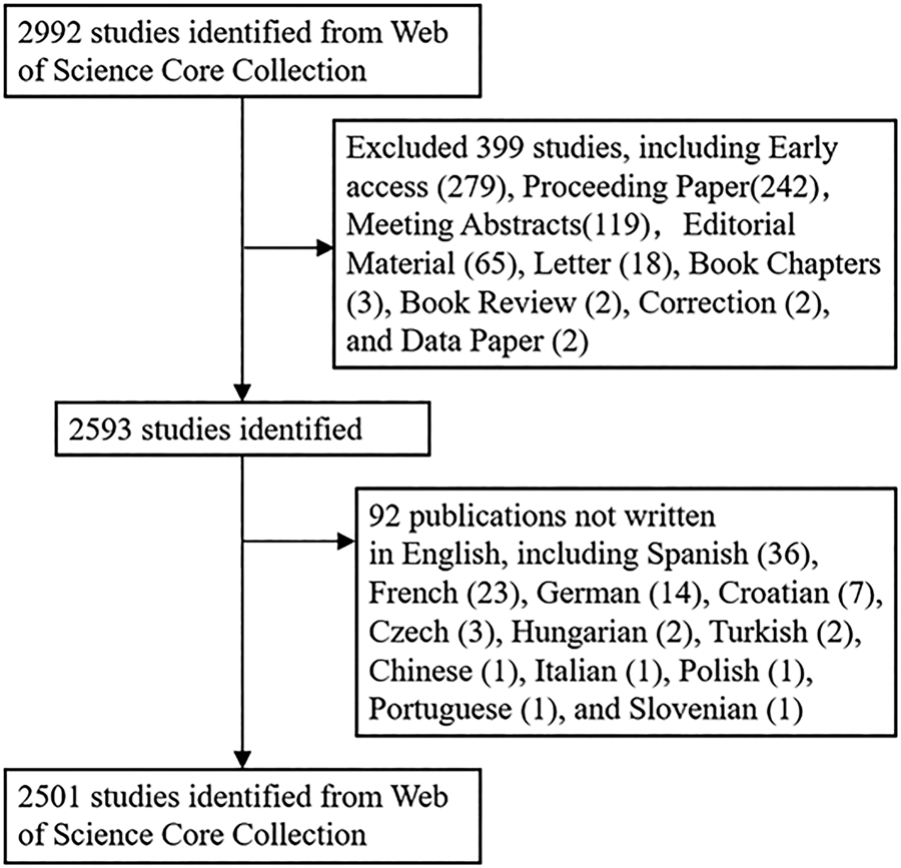
Literature search process.

A comprehensive literature review was conducted using the Web of Science Core Collection (WoSCC) database. The search query employed was: (TS = (neonat* OR newborn)) AND TS = (EEG OR electroencephalogram* OR electroencephalography). The search was restricted to “articles” and “reviews” as the relevant document types. The inclusion criteria stipulated that the studies must be in English and any redundant entries were subsequently removed. The search period spanned from January 1, 2004 to December 31, 2022, yielding a total of 2501 studies. Figure 1 illustrates the search strategy.

### 2.2 Data analysis

VOSviewer (RRID:SCR_023516) (ver. 1.6.19) is a bibliometric tool used to extract significant insights from extensive publication datasets (van Eck & Waltman, 2010). It is commonly applied to develop networks of collaboration, co‐citation, and co‐occurrence (Pan et al., 2018; Yeung & Mozos, 2020). In this research, VOSviewer facilitated the analysis of countries and institutions, evaluation of journals and co‐cited journals, examination of authors and co‐cited authors, and exploration of keyword co‐occurrence patterns. In the visual representations generated by VOSviewer, each node represents an entity, such as a country, institution, journal, or author. The size and color of a node denote the volume and type of these entities, respectively, whereas the thickness of the lines connecting nodes indicates the level of collaboration or co‐citation among them (Wu et al., 2021; Zhang et al., 2020).

CiteSpace (RRID: SCR_025121) (ver. 6.2.4), developed by Chen C, is another bibliometric visualization tool (Synnestvedt et al., 2005). In this study, CiteSpace was utilized to present the journal dual‐map overlay and to conduct reference analysis through citation bursts.

Bibliometrix (RRID:SCR_023744) (ver. 4.1.2) was employed to analyze thematic evolution and to create a global distribution map of publications on neonatal sleep EEG (Aria & Cuccurullo, 2017). Data on journal quartiles and impact factors were obtained from the Journal Citation Reports 2022.

Additionally, a quantitative analysis of the publications was conducted using Microsoft Office Excel 2021.

## 3 RESULTS

### 3.1 Quantitative analysis of publications

**FIGURE 2:**
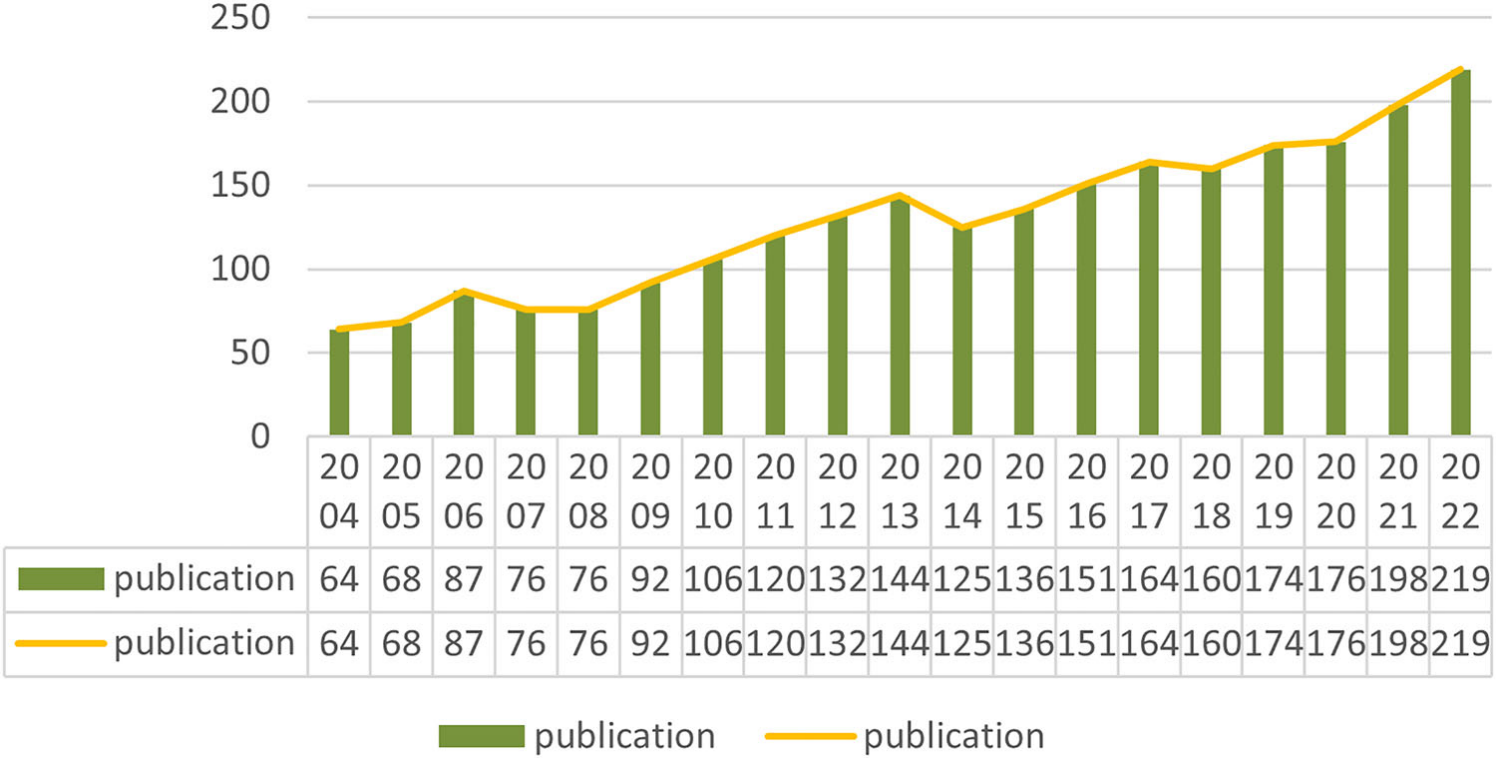
Annual output of research of neonatal EEG.

Based on our search criteria, a total of 2501 studies related to sleep EEG in neonates were identified from 2004 to 2022, comprising 2221 “articles” and 280 “reviews.” The annual publication growth rate remained relatively stable, with an average yearly increase of eight articles. As illustrated in Figure 2, there is a noticeable rise in the number of publications from 64 to 219 articles annually over the 2004 to 2022 period. This trend underscores the sustained research interest in neonatal EEG within the evolving landscape of scientific inquiry.

### 3.2 Country and organization assessment

**FIGURE 3:**
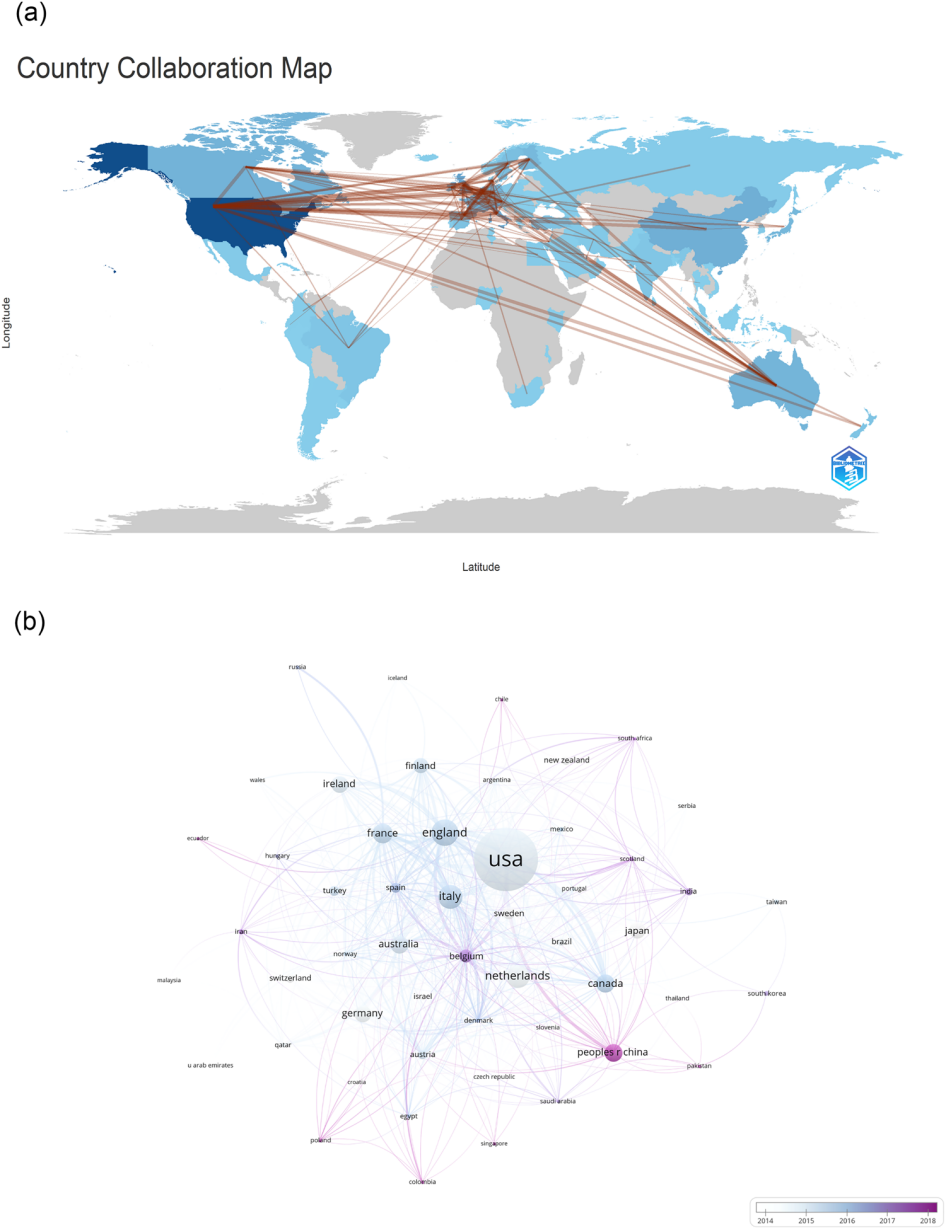
The geographical distribution (a) and visualization of countries (b) on research of neonatal EEG.

The collected publications stem from 79 countries and 2639 organizations. The top 10 contributing countries include notable representation from North America, Europe, and Asia, with Europe dominating (*n* = 6), as delineated in Table 1. The United States leads in publication volume (*n* = 765, 30.6%), followed by England (*n* = 242, 9.7%), Italy (*n* = 213, 8.5%), and the Netherlands (*n* = 208, 8.3%), highlighting the substantial contribution from the United States (30.6%). Data from 52 countries, each with at least five publications, were further analyzed to construct a collaborative network based on publication count and inter‐country collaborations (Figure 3). This network reveals robust international collaboration, with the United States notably collaborating with England, France, and Canada, while England shows significant cooperation with Ireland, the Netherlands, and Belgium.

**TABLE 1:** Top 10 countries and organizations on research of neonatal EEG.

Rank	Country	Counts	Institution	Counts
1	The United States (North America)	765 (30.6%)	University of Helsinki (Finland)	102 (4.1%)
2	England (Europe)	242 (9.7%)	University College London (England)	76 (3.0%)
3	Italy (Europe)	213 (8.5%)	University College Cork (Ireland)	67 (2.7%)
4	Netherlands (Europe)	208 (8.3%)	University of Pennsylvania (The United States)	66 (2.6%)
5	France (Europe)	168 (6.7%)	University of California, San Francisco (The United States)	65 (2.6%)
6	Canada (North America)	151 (6.0%)	University Medical center Utrecht (Holland)	51 (2.0%)
7	the People's Republic of China (Asia)	145 (6.0%)	Stanford University (The United States)	49 (2.0%)
8	Australia (Oceania)	143 (5.7%)	The University of Queensland (Australia)	49 (2.0%)
9	Germany (Europe)	135 (5.4%)	The George Washington University (The United States)	47 (1.9%)
10	Ireland (Europe)	133 (5.3%)	Children's Hospital of Philadelphia (The United States)	46 (1.8%)

**FIGURE 4:**
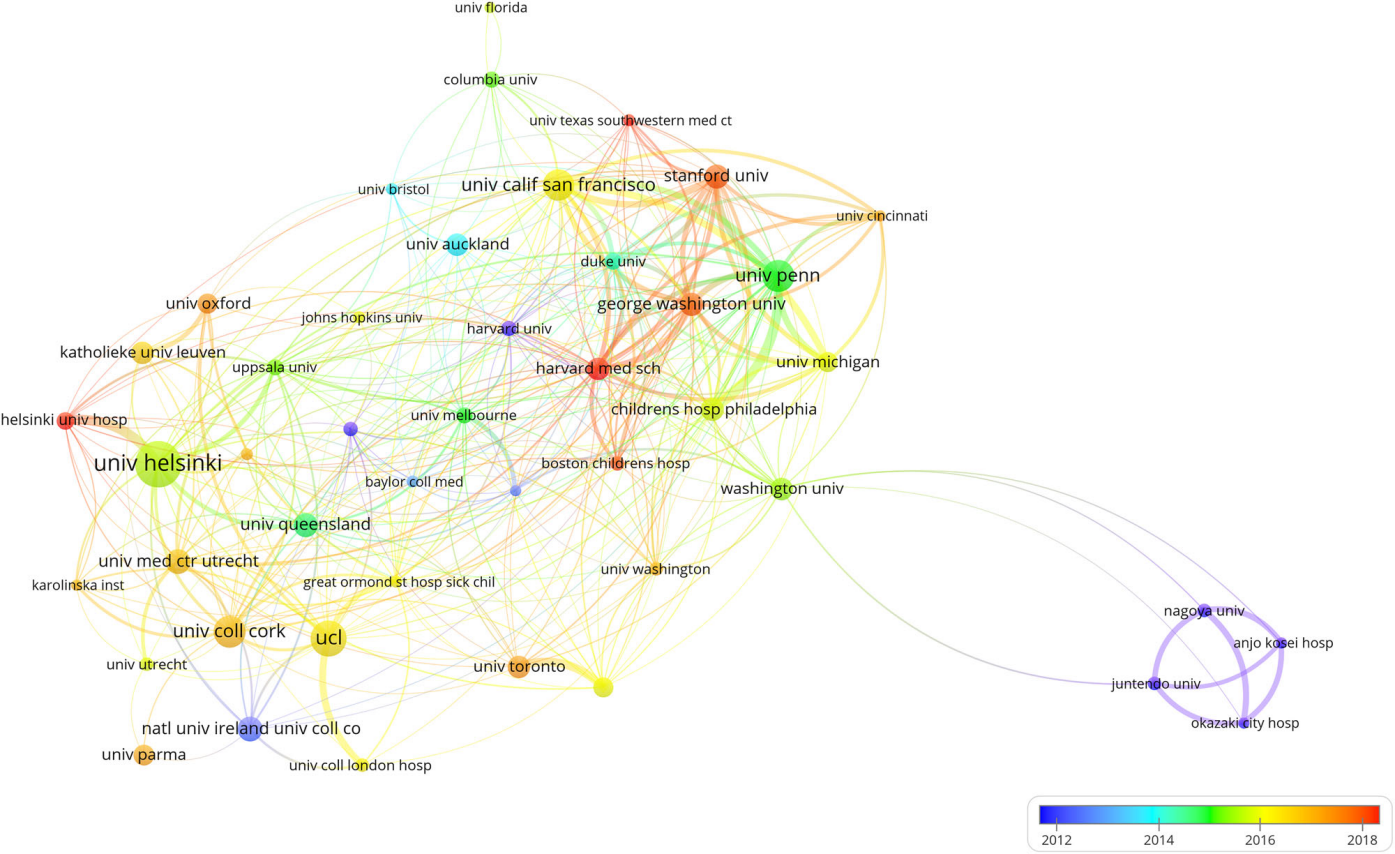
Organizations on research of neonatal EEG.

The leading 10 organizations are distributed across six countries, half of which are in the United States. The top contributors include the University of Helsinki (*n* = 102, 4.1%), University College London (*n* = 76, 3.0%), and University College Cork (*n* = 67, 2.7%). An analysis of 45 organizations, each with a minimum of 20 publications, facilitated the creation of an inter‐institutional cooperative network (Figure 4), showcasing significant collaborations among institutions like the University of Pennsylvania, University of California, San Francisco, and The George Washington University, paralleled by active cooperation between the University of Helsinki and Helsinki University Central Hospital. Simultaneously, it has been observed that the bulk of publications from the United States predominantly concentrated around the year 2014. However, the period following 2017 has witnessed a surge in the publication of papers by researchers in China and Belgium.

### 3.3 Analysis of journals and co‐cited journals

**FIGURE 5:**
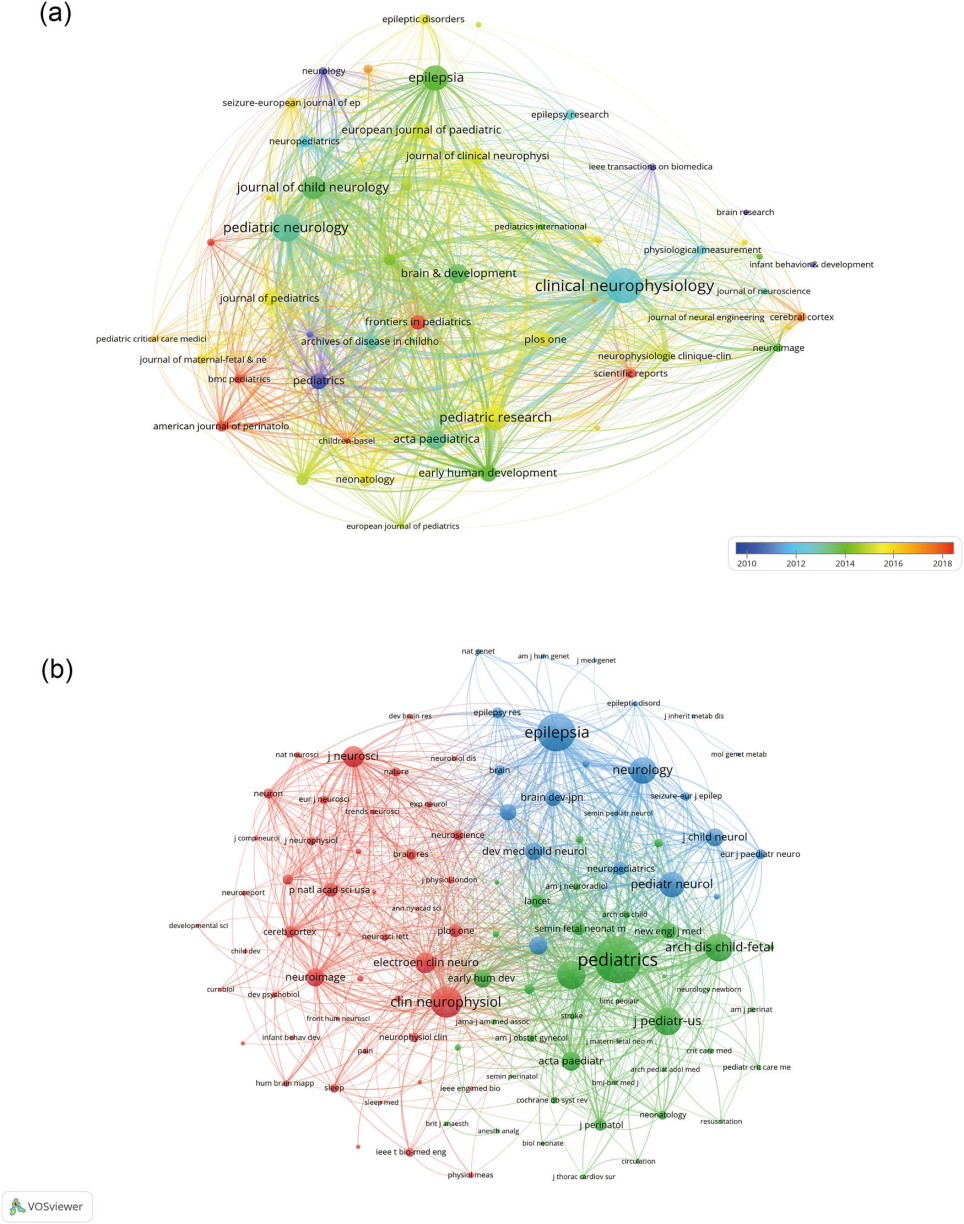
Journals (a) and co‐cited journals (b) on research of neonatal EEG.

Research on neonatal sleep EEG was published across 544 journals, with *Clinical Neurophysiology* being the most prolific (*n* = 115, 4.6%), followed by *Pediatric Neurology* (*n* = 84, 3.4%), *Epilepsia* (*n* = 74, 3.0%), and *Pediatric Research* (*n* = 11, 2.9%). The top 15 journals include *Pediatrics* with the highest impact factor (IF = 8.0), succeeded by *Epilepsia* (IF = 5.6) and *Journal of Pediatrics* (IF = 5.1). A network mapping of 53 journals, each with at least 10 relevant publications, was conducted to visualize the journal interconnectivity (Figure 5a).

Table 2 shows that among the top 15 co‐cited journals, two were cited over 3000 times, with *Pediatrics* leading (co‐citation = 4915), followed by *Epilepsia* (co‐citation = 3622) and *Clinical Neurophysiology* (co‐citation = 2766). A co‐citation network was mapped for journals with at least 150 co‐citations, illustrating the citation dynamics within the field Figure 5b.

**TABLE 2:** Top 15 journals and co‐cited journals for research of neonatal EEG.

Rank	Journal	Count	IF (2022)	Q	Co‐cited Journal	Co‐citation	IF	Q
1	*Clinical Neurophysiology*	115 (4.6%)	4.7	Q1	*Pediatrics*	4915	8.0	Q1
2	*Pediatric Neurology*	84 (3.4%)	3.8	Q1	*Epilepsia*	3622	5.6	Q1
3	*Epilepsia*	74 (3.0%)	5.6	Q1	*Clinical Neurophysiology*	2766	4.7	Q2
4	*Pediatric Research*	72 (2.9%)	3.6	Q1	*Archives of Disease in Childhood‐Fetal and Neonatal Edition*	2412	4.4	Q1
5	*Journal of Child Neurology*	66 (2.6%)	1.9	Q2	*Pediatric Research*	2373	3.6	Q1
6	*Acta Paediatrica*	53 (2.1%)	3.8	Q1	*Journal of Pediatrics*	2352	5.1	Q1
7	*Brain & Development*	53 (2.1%)	1.7	Q3	*Neurology*	2188	10.1	Q1
8	*Pediatrics*	43 (1.7%)	8.0	Q1	*Pediatric Neurology*	2097	3.8	Q1
9	*Early Human Development*	43 (1.7%)	2.5	Q3	*Journal of Neuroscience*	1614	5.3	Q1
10	*European Journal of Paediatric Neurology*	43 (1.7%)	3.1	Q1	*Electroencephalography Clinical Neurophysiology*	1565	3.3	Q1
11	*Journal of Clinical Neurophysiology*	41 (1.6%)	2.4	Q2	*Acta Paediatrica*	1410	3.8	Q1
12	*Journal of Pediatrics*	38 (1.5%)	5.1	Q1	*Neuroimage*	1283	5.7	Q1
13	*PLoS One*	36 (1.4%)	3.7	Q2	*Journal of Child Neurology*	1279	1.9	Q2
14	*Frontiers in Pediatrics*	36 (1.4%)	2.6	Q2	*Journal of Clinical Neurophysiology*	1274	2.4	Q2
15	*Archives of Disease in Childhood‐Fetal and Neonatal Edition*	34 (1.4%)	4.4	Q1	*Developmental Medicine and Child Neurology*	1266	3.8	Q2

**FIGURE 6:**
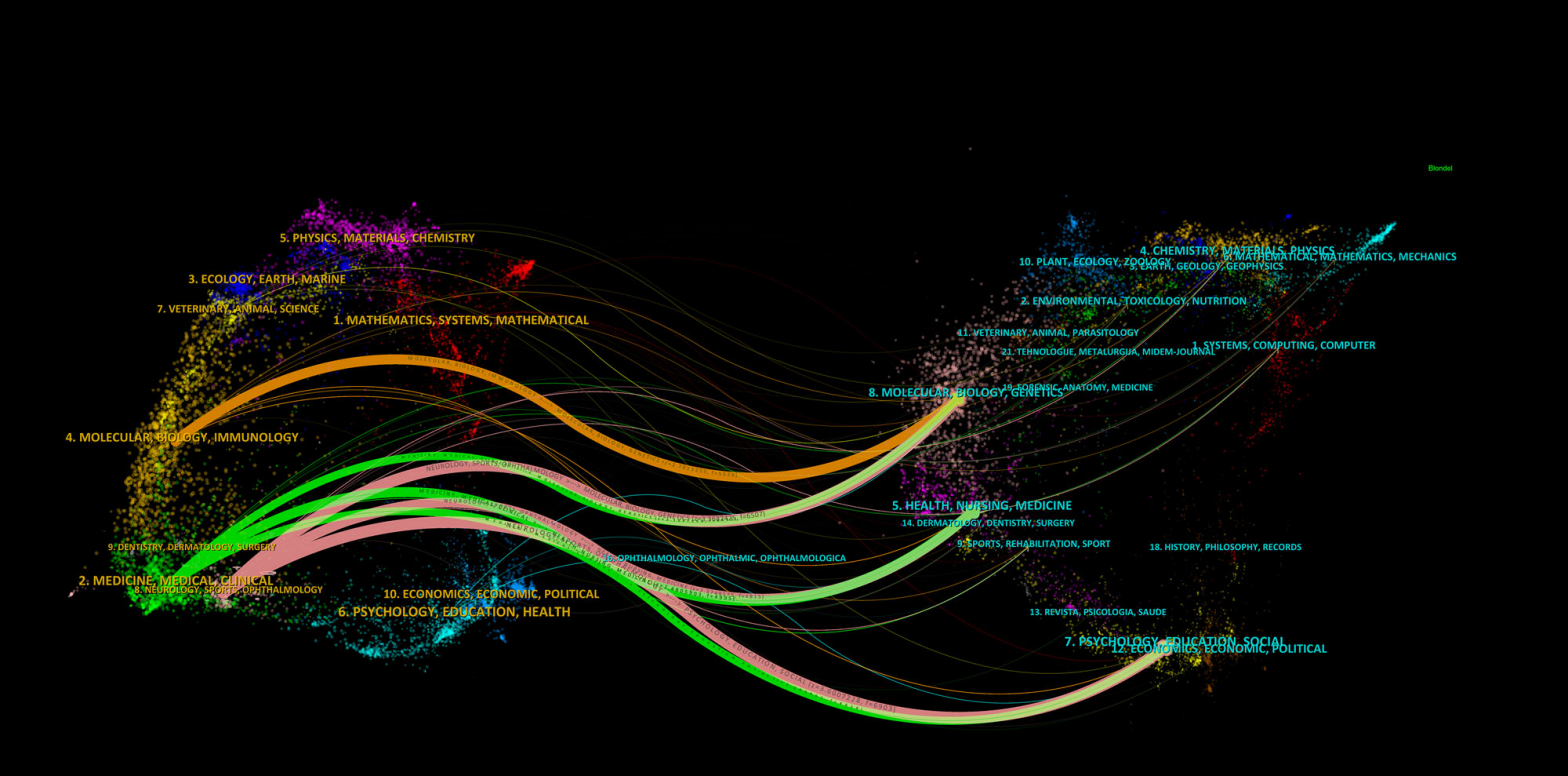
The dual‐map overlay of journals on research of neonatal EEG.

The dual‐map overlay of journals (Figure 6) demonstrates the citation flow between citing and co‐cited journals (Hellström‐Westas et al., 1995), highlighting the primary disciplines of the citing and cited bodies of literature. A large portion of the citing publications were distributed across the domains of Molecular Biology, Genetics, Health, Nursing, Medicine, Psychology, and Education. Conversely, the bulk of cited articles were chiefly situated within the disciplines of Molecular Biology, Immunology, Medicine, and Clinical Medicine.

### 3.4 Analysis of authors and co‐cited authors

**FIGURE 7:**
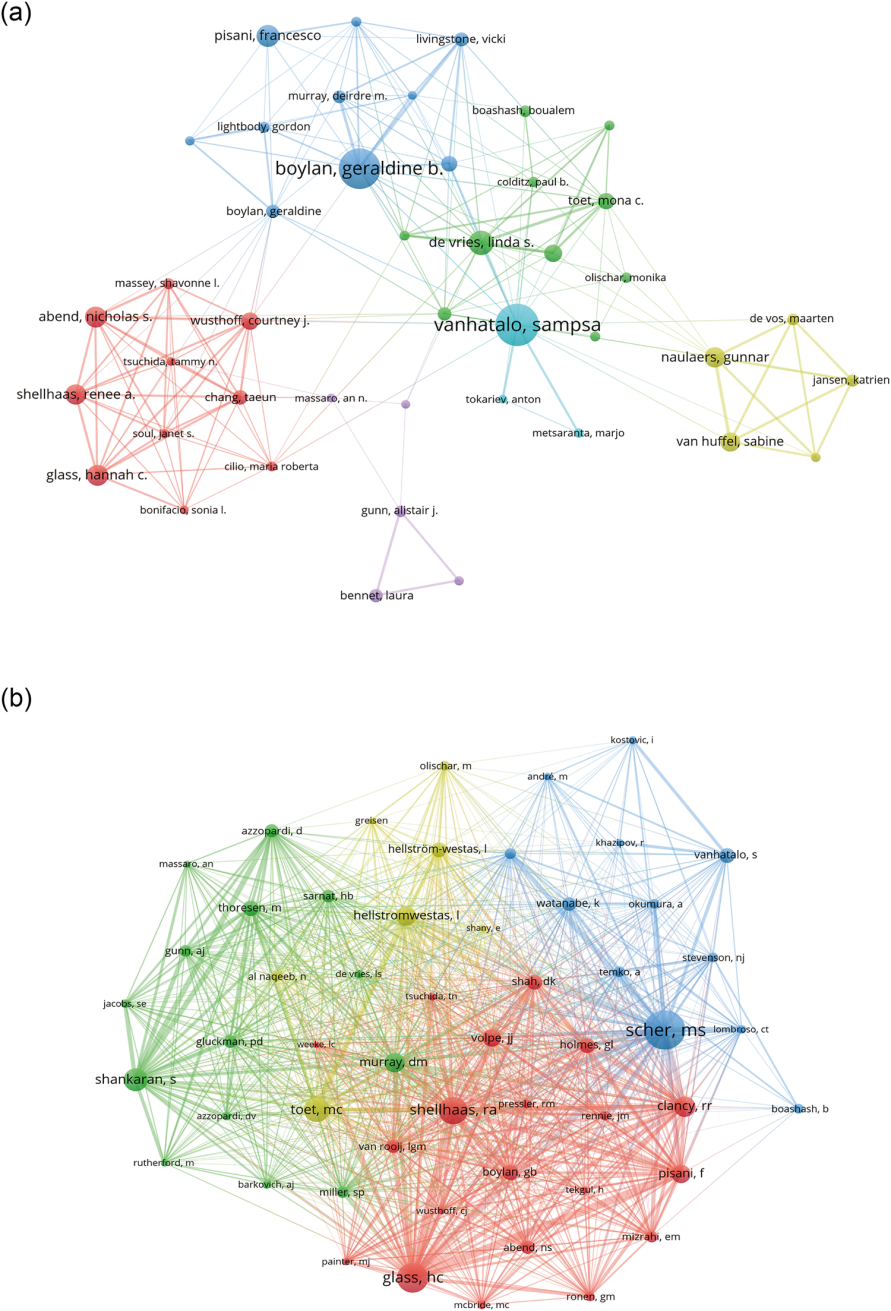
Authors (a) and co‐cited authors (b) on research of neonatal EEG.

A total of 2116 authors have contributed to the neonatal sleep EEG research body. The top 10 authors each published over 30 papers (Table 3). A collaborative network of authors with fifteen or more publications was visualized in Figure 7a. Vanhatalo emerged as the most prominent node, reflecting the extensive volume of his contributions, showing key contributors like Vanhatalo and Geraldine B. Boylan at the forefront. Notable collaborations were observed, for example, between Gunnar Naulaers and Katrien Jansen.

**TABLE 3:** Top 10 authors and co‐cited authors on research of neonatal EEG.

Rank	Authors	Count	Co‐cited authors	Citations
1	Sampsa Vanhatalo	71	Mark Steven Scher	890
2	Geraldine B. Boylan	68	Hannah C. Glass	654
3	Linda S. De Vries	41	Renee A. Shellhaas	600
4	Francesco Pisani	37	Mona C. Toet	523
5	Hannah C. Glass	35	Seetha Shankaran	492
6	Nicholas S. Abend	35	Robert R. Clancy	437
7	Renee A. Shellhaas	34	Lena Hellstrom‐Westas	414
8	Gunnar Naulaers	34	Deirdre M. Murray	408
9	Sabine Van Huffel	32	Francesco Pisani	386
10	Courtney J. Wusthoff	30	Joseph J. Volpe	363

Among the 33,123 co‐cited authors, four were co‐cited more than 500 times, with Mark Steven Scher leading (*n* = 890). A co‐citation network for authors with at least 120 co‐citations was created (Figure 7b), revealing active collaborations within the community, such as between Hannah C. Glass and Renee A. Shellhaas.

### 3.5 Co‐cited references

**FIGURE 8:**
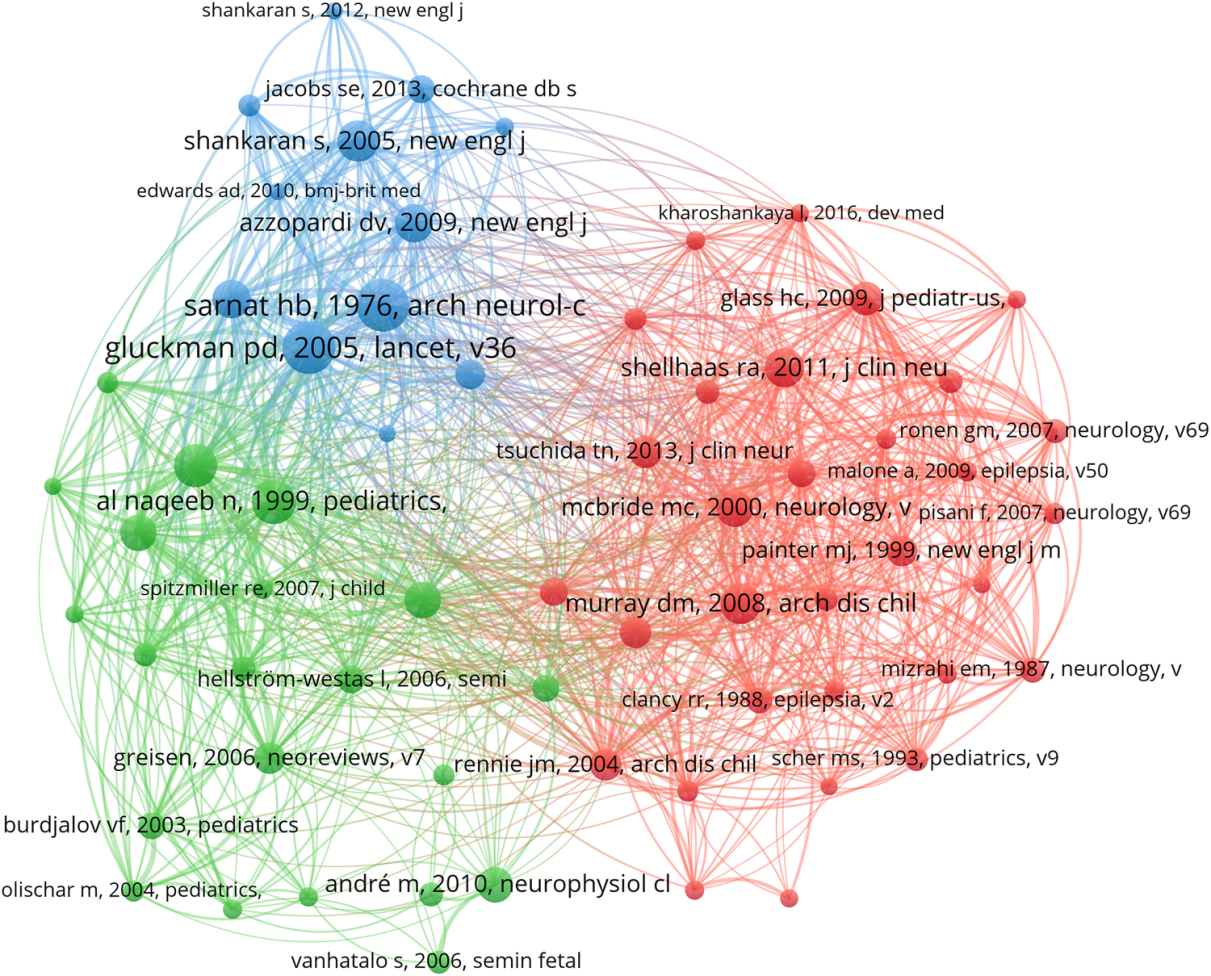
Co‐cited references on research of sleep EEG in neonates.

In the last two decades, there have been 52,202 co‐cited references in the field of sleep EEG research in neonates. Among the top 10 co‐cited references (Table 4), each has been co‐cited at least 151 times, with two references receiving more than 239 co‐citations (Gluckman et al., 2005; Sarnat & Sarnat, 1976). We selected references with 60 or more co‐citations to develop the co‐citation network map (Figure 8). According to Figure 8, “Gluckman Pd, 2005, Lancet” demonstrated active co‐citation links with “Sarnat Hb, 1976, Arch Neurol‐chicago,” “Al naqeeb N, 1999, Pediatrics,” and “Toet Mc, 1999, Arch Dis Child‐fetal,” among others.

**TABLE 4:** Top 10 co‐cited references on research of neonatal EEG.

Rank	Co‐cited reference	Citations	Type
1	Gluckman Pd, 2005, Lancet, V365, P663 (Gluckman et al., 2005)	239	Article
2	Sarnat Hb, 1976, Arch Neurol‐chicago, V33, P696 (Sarnat & Sarnat, 1976)	239	Article
3	Al naqeeb N, 1999, Pediatrics, V103, P1263 (al Naqeeb et al., 1999)	192	Article
4	Toet Mc, 1999, Arch Dis Child‐fetal, V81, Pf19 (Toet et al., 1999)	188	Article
5	Shankaran S, 2005, New Engl J Med, V353, P1574 (Shankaran et al., 2005)	175	Article
6	Murray Dm, 2008, Arch Dis Child‐fetal, V93, Pf187 (Murray et al., 2008)	170	Article
7	Shellhaas Ra, 2011, J Clin Neurophysiol, V28, P611 (Shellhaas et al., 2011)	167	Article
8	Azzopardi Dv, 2009, New Engl J Med, V361, P1349 (Azzopardi et al., 2009)	162	Article
9	Thoresen M, 2010, Pediatrics, V126, Pe131 (Thoresen et al., 2010)	160	Article
10	Hellstromwestas L, 1995, Arch Dis Child‐fetal, V72, Pf34 (Hellström‐Westas et al., 1995)	151	Article

### 3.6 Reference with citation bursts

**FIGURE 9:**
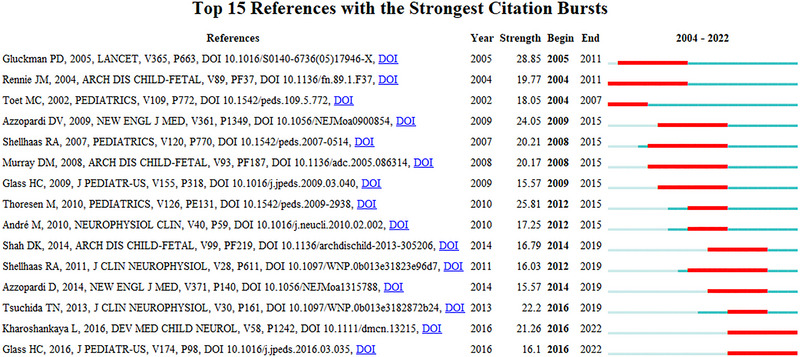
Top 15 references with strong citation bursts. A red bar indicates high citations in that year.

Citation bursts refer to references that receive a significant number of citations within a specific timeframe, indicating a high level of academic interest. In this analysis, CiteSpace identified 15 references with notable citation bursts (Figure 9), where each bar represents a year and the red bar indicates a period of significant citation activity (Azzopardi et al., 2009). Citation bursts were observed as early as 2004 and as recent as 2022. The strongest citation burst (strength = 28.85) was attributed to “Selective head cooling with mild systemic hypothermia after neonatal encephalopathy: multicentre randomised trial” by Peter D. Gluckman et al., which garnered extensive citations from 2005 to 2011. The next highest citation burst (strength = 25.81) was for “Non‐expert use of the cerebral function monitor for neonatal seizure detection” by J. M. Rennie et al., with a notable increase in citations from 2012 to 2015.

These 15 references had burst strengths ranging from 15.57 to 28.85, and their burst durations varied from 4 to 7 years. Table 5 organizes the primary research topics of these 15 references, corresponding to their order in Figure 9.

**TABLE 5:** The main research contents of the 15 references with strong citations bursts.

Rank	Strength	Main research content
1	28.85	The efficacy of delayed head cooling in neonates with encephalopathy, exhibiting less severe aEEG abnormalities, yet ineffectiveness in those with the most severe aEEG alterations. (Gluckman et al., 2005)
2	19.77	Cerebral Function Monitor (CFM) to be less effective, indicating that EEG should precede CFM for characterizing neonatal seizures, with CFM potentially serving for long‐term monitoring. (Rennie et al., 2004)
3	18.05	CFM to be a reliable tool for monitoring background activity and ictal activity. Recommend to use CFM for monitoring, supplemented by standard EEG for ambiguous cases or suspected epileptiform activity. (Toet et al., 2002)
4	24.05	Hypothermic therapy in newborns with asphyxial encephalopathy did not reduce death or severe disability but improved neurological outcomes among survivors, without significant adverse events related to cooling. (Azzopardi et al., 2009)
5	20.21	Even experienced physicians struggle to detect many neonatal seizures using aEEG, especially those that are brief, infrequent, or low in amplitude. (Shellhaas et al., 2007)
6	20.17	Underscoring the importance of continuous video‐EEG monitoring for accurate detection and management in identifying neonatal seizures. (Murray et al., 2008)
7	15.57	Neonatal seizures are linked to poorer motor and cognitive outcomes in infants with hypoxia‐ischemia. (Glass et al., 2009)
8	25.81	Early aEEG patterns effectively predict outcomes in normothermia‐treated infants with perinatal asphyxia, but not hypothermia, with normal aEEG background and sleep‐wake cycling being crucial predictive indicators. (Thoresen et al., 2010)
9	17.25	Developmental features and glossary of electroencephalography in infants. (André et al., 2010)
10	16.79	Newborns with HIE undergoing TH, the burden of electrographic seizures is independently associated with the severity of brain injury on MRI. (Shah et al., 2014)
11	16.03	Outlines preferred practices for long‐term, conventional EEG monitoring in high‐risk neonates under 48 weeks postmenstrual age. (Shellhaas et al., 2011)
12	15.57	Moderate hypothermia treatment for newborns with asphyxial encephalopathy led to better neurocognitive outcomes at 6 to 7 years of age. (Azzopardi et al., 2014)
13	22.2	Continuous video EEG monitoring is crucial for assessing brain function and detecting seizures without clinical signs in neonates. (Tsuchida et al., 2013)
14	21.26	A high seizure burden significantly increased the risk of adverse outcomes, regardless of HIE severity or hypothermia treatment. (Kharoshankaya et al., 2016)
15	16.1	Newborns monitored with cEEG, seizures were mostly caused by brain injuries, with a high number of seizures leading to worse health outcomes. (Glass et al., 2016)

### 3.7 Hotspots and frontiers

Keyword co‐occurrence analysis is a tool for swiftly identifying research hotspots within a field. Table 6 lists the top 20 high‐frequency keywords related to sleep EEG research in neonates. Notably, “hypoxic‐ischemic encephalopathy (HIE)” and “seizures” were among the most frequent, each occurring over 300 times, highlighting the main research directions in this domain.

**TABLE 6:** Top 20 keywords on research of neonatal EEG.

Rank	Keywords	Counts	Rank	Keywords	Counts
1	EEG	696	11	Newborn	219
2	Infants	380	12	Brain	214
3	Hypoxic‐ischemic encephalopathy	369	13	Hypothermia	192
4	Electroencephalography	336	14	Neonatal encephalopathy	185
5	Seizures	311	15	Amplitude‐integrated electroencephalography	184
6	Epilepsy	299	16	Brain injury	179
7	Neonatal seizures	274	17	Therapeutic hypothermia	174
8	Children	266	18	Electroencephalogram	173
9	Preterm infants	226	19	Encephalopathy	160
10	Newborns	222	20	Preterm	152

**FIGURE 10:**
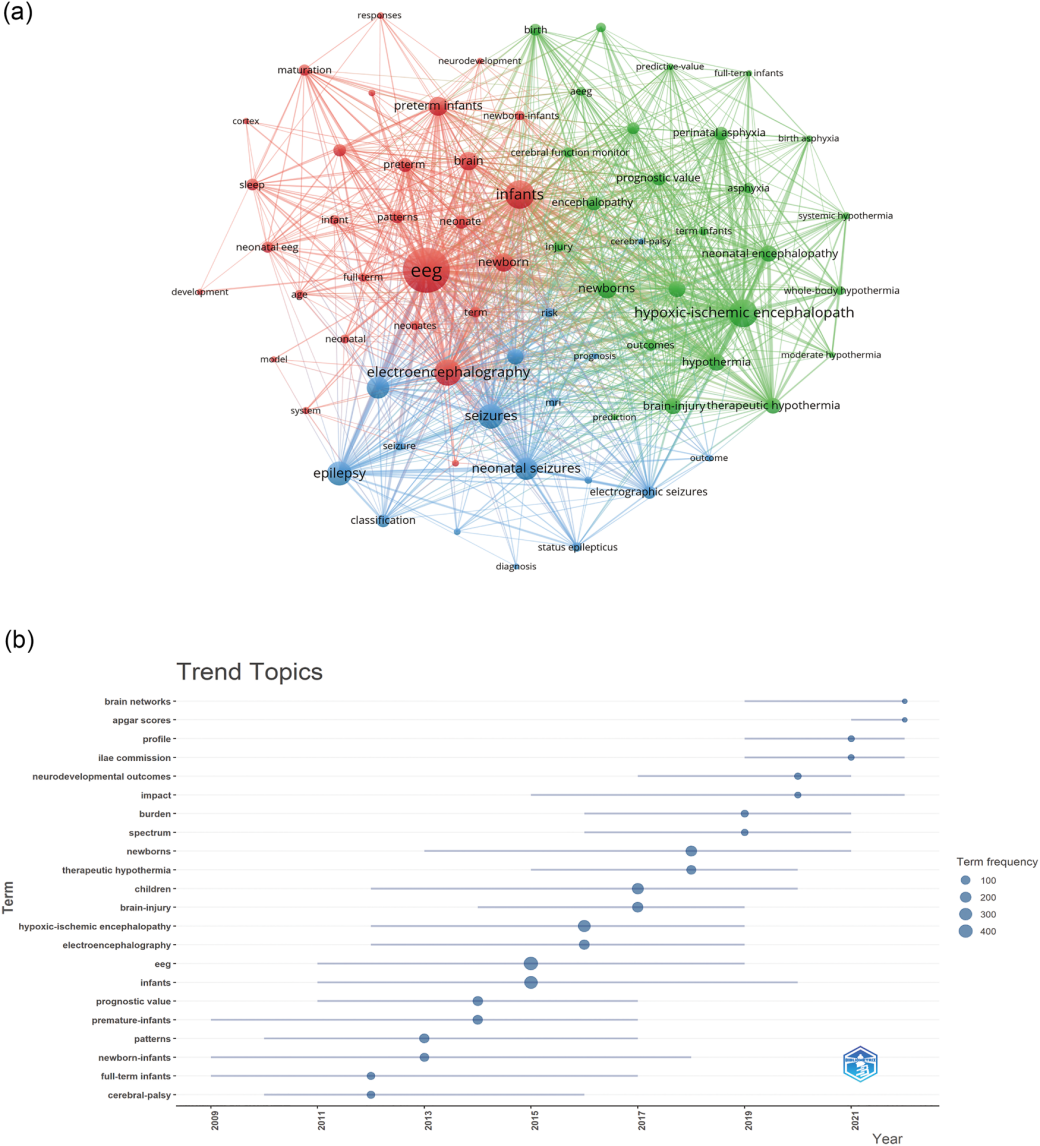
Keyword cluster analysis (a) and trend topic analysis (b).

A refined analysis was conducted for keywords appearing fifty times or more, leading to a cluster analysis via VOSviewer (Figure 10a). The line thickness between nodes signifies the association strength between keywords. Figure 10a reveals three distinct clusters: the red cluster includes keywords like “EEG,” “infants,” and “brain”; the green cluster contains “hypoxic‐ischemic encephalopathy,” “brain injury,” and “hypothermia”; and the blue cluster comprises “seizures” and “epilepsy.”

The keyword trend analysis (Figure 10b) provides insights into the shifting research focus over time. From 2009 to 2021, the emphasis was predominantly on “EEG” and “infants,” with recurrent keywords such as “hypoxic‐ischemic encephalopathy,” “children,” and “brain injury.” After 2017, the research expanded toward exploring EEG changes related to HIE prognosis, highlighted by frequent terms like “therapeutic hypothermia (TH),” “neurodevelopmental outcomes,” and “International League Against Epilepsy (ILAE) commission.” Notably, “brain network” emerged as a significant term from 2021 to 2022, suggesting its potential as a current research hotspot in neonatal EEG studies.

## 4 DISCUSSION

The United States stands out as a leading nation in neonatal EEG research, with approximately 30% of the top 10 pioneering research organizations located there, followed by England and Italy, each hosting two organizations, accounting for 9.7% and 8.5% respectively. Notable collaborative patterns are observed among four countries: the United States, England, France, and Italy. Additionally, England demonstrates significant collaborative efforts with Ireland, Belgium, and the Netherlands.

In terms of research institutions, a productive collaboration is evident among the University of Pennsylvania, Stanford University, University of California, San Francisco, and The George Washington University. On the contrary, institutions like Nagoya University, Anjo Kosei Hospital, Juntendo University, and Okazaki City Hospital, while being prolific in publication output, show limited collaboration with other entities. While inter‐country collaborations are present, the depth and breadth of cooperation between organizations, particularly between those in Asia and Europe, seem to be less than optimal.

*Clinical Neurophysiology* (IF = 4.7, Q1) emerges as a leading journal in neonatal EEG research, with *Pediatrics* (IF = 8.0, Q1) having the highest impact factor, followed by *Epilepsia* (IF = 5.6). A review of co‐cited journals reveals that most are high‐impact Q1 journals, highlighting their significant global recognition and contribution to neonatal EEG research. The predominant research outputs are published in journals encompassing Molecular Biology, Genetics, Health, Nursing, Medicine, Psychology, and Education, reflecting a broad spectrum of clinical and basic research.

From an author‐focused perspective, Sampsa Vanhatalo leads with 71 publications, followed by Geraldine B. Boylan and Linda S. De Vries with 68 and 41 papers respectively. Sampsa Vanhatalo's highly cited review underscores the importance of neonatal EEG in monitoring preterm infants' brain functions. It advocates for a simplified, pattern‐based framework to enhance EEG interpretation, enable automated analysis, and foster connections between basic science and clinical practice (Vanhatalo & Kaila, 2006).

In the context of co‐cited authors, Mark Steven Scher is the most cited, with 890 citations, followed by Hannah C. Glass (*n* = 654) and Renee A. Shellhaas (*n* = 650). Mark Steven Scher's research focuses on continuous EEG monitoring in neonates and clinical neurophysiology, crucial for developing guidelines and standardized terminology to improve neonatal brain dysfunction diagnosis and management (Shellhaas et al., 2011; Tsuchida et al., 2013). Hannah C. Glass has built upon Scher's work, significantly impacting neonatal neurology, particularly in enhancing seizure management and monitoring in HIE (Glass et al., 2016; Nash et al., 2011).

### 4.1 Knowledge base

A co‐cited reference, recognized when multiple publications concurrently cite it, is indicative of foundational knowledge within a specific field (Chen, 2006). In this study, we selected the top 10 co‐cited references to delineate the research foundation of neonatal EEG. These pivotal documents predominantly focus on neonatal hypoxic‐ischemic encephalopathy (HIE), reflecting the research trajectory from clinical symptom descriptions and prognosis evaluations to treatment methodologies, encapsulating the gamut from descriptive studies to randomized controlled trials. The second most cited document, dating back to 1976, delves into the clinical and electroencephalographic assessment of three distinct stages of postanoxic encephalopathy in neonates (Sarnat & Sarnat, 1976). The 3rd, 4th, and 10th documents, from 1999 and 1995, underscore the utility of aEEG in prognostication and early intervention postbirth asphyxia (al Naqeeb et al., 1999; Hellström‐Westas et al., 1995; Toet et al., 1999). Documents ranked 1, 5, and 8, authored by Gluckman Pd, Shankaran S, and Azzopardi Dv, respectively (Azzopardi et al., 2009; Gluckman et al., 2005; Shankaran et al., 2005), explore treatments for asphyxial encephalopathy, noting improved neurological outcomes in survivors despite not significantly reducing death or severe disability rates.

Notably, Professor Gluckman Pd led a seminal and frequently co‐cited 2005 study, contributing significantly to this field. The 9th document, from 2009, evaluates different cooling methods for newborns with perinatal asphyxia, highlighting servo‐controlled whole‐body cooling's efficacy in maintaining target body temperatures (Hoque et al., 2010).

The sixth article, by Murray Dm in 2008, points out the limitations of clinical diagnoses in neonatal seizure detection, emphasizing continuous video‐EEG monitoring's role in accurate detection and management (Murray et al., 2008). In 2011, guidelines for neonatal brain electrical monitoring were introduced by Shellhaas Ra, offering recommendations for long‐term EEG monitoring in high‐risk neonates below 48 weeks postmenstrual age, addressing the challenges and diverse clinical needs for EEG monitoring and interventions (Shellhaas et al., 2011).

In summation, the 10 most co‐cited references concentrate on HIE, seizures, aEEG techniques, and neurological development monitoring, collectively forming the cornerstone of research in the neonatal EEG and neurodevelopment sector.

### 4.2 Hotspots and frontiers

References with citation bursts highlight emerging research areas that have garnered significant attention recently (Miao et al., 2018). Table 5 analyzes the primary content of references with pronounced citation bursts, revealing a shift toward examining EEG characteristics in postanoxic encephalopathy and neonatal seizures. There is an observable trend from focusing on aEEG to integrating magnetic resonance imaging (MRI) for monitoring neonatal encephalopathy prognosis.

Beyond citation bursts, keyword analysis offers insights into the field's evolving hotspots. Excluding common terms like EEG, newborn, and HIE, Table 6 predominantly features keywords such as preterm infants, epilepsy, aEEG, therapeutic hypothermia, and brain injury. The keyword clustering and trend analysis (Figure 10) suggest that neonatal EEG research predominantly encompasses aspects of preterm infant care, epilepsy, aEEG utility, therapeutic hypothermia interventions, and brain injury assessments.

### 4.3 Cerebral function monitor

The term “cerebral function monitor (CFM)” predominantly refers to a single‐channel aEEG, now extensively utilized in neonatal intensive care units (NICUs) (Boylan et al., 2013). Research as early as 1999 established aEEG's reliability and accuracy in assessing neonatal encephalopathy, highlighting its predictive value for neurodevelopmental outcomes through the identification of normal and abnormal aEEG patterns (al Naqeeb et al., 1999; Toet et al., 1999). Further studies have corroborated aEEG's high concordance with standard EEG in identifying severely abnormal patterns and seizures (Toet et al., 1999), affirming its efficacy in monitoring neonates with hypoxic‐ischemic encephalopathy (HIE) or suspected seizures. It demonstrates notable reliability in detecting background activity and ictal events compared to standard EEG. However, challenges persist in using aEEG from a single EEG channel for neonatal seizure detection. Despite its theoretical high sensitivity for seizure detection, actual detection rates by clinicians show considerable variability, influenced by factors such as seizure characteristics, medication effects, and the clinician's familiarity with aEEG (Omidvarnia et al., 2014; Shellhaas et al., 2007).

Studies indicate that nonexperts employing aEEG in NICUs to detect neonatal seizures have limited accuracy, failing to identify approximately half of the seizures compared to concurrent standard EEG (Malone et al., 2009; Osredkar et al., 2005; Rennie et al., 2004; Shah et al., 2008). Interobserver agreement remains low, suggesting that while CFM may miss less severe or focal seizures, it remains valuable for long‐term monitoring postinitial EEG‐based seizure diagnosis and characterization. This underscores the vital need for dependable continuous neurological monitoring in neonates to ensure precise diagnosis and treatment, aiming to reduce the risk of overlooked seizures or the administration of unnecessary medications for nonseizure‐related movements (Malone et al., 2009).

In light of these findings, some researchers advocate for a combined approach, integrating aEEG with 2‐channel EEG, to enhance the reliability of monitoring electrical seizures in neonates at risk (Shah et al., 2008). Another proposed method involves using aEEG alongside sleep‐wake cycling (SWC) indicators. The onset of SWC varies significantly among different HIE severities and is delayed in newborns with seizures, providing valuable insights for improved monitoring and intervention strategies (Osredkar et al., 2005).

### 4.4 Brain network

The investigation of brain networks through neonatal EEG predominantly targets the establishment of an automated epilepsy recognition system. Early efforts in this field utilized time‐frequency analysis to address the intricate and nonstationary characteristics of neonatal seizure signals (Hassanpour et al., 2004). By evaluating the distribution functions of singular vectors derived from the EEG signals’ time‐frequency distribution, this technique effectively distinguishes between seizure and nonseizure states. These functions are subsequently employed to train a neural network, facilitating precise seizure detection. Subsequent developments saw the incorporation of filtering, artifact identification, feature extraction, and neural network classification, evolving into a knowledge‐driven decision‐making framework using backpropagation neural networks (Aarabi et al., 2006; Aarabi et al., 2007). The adoption of deep convolutional neural networks (CNNs) and random forests further advanced this methodology, enhancing feature selection and classification directly from raw multi‐channel EEG data (Ansari et al., 2019), significantly reducing the false positive rate from 1.55 to 0.9 per hour.

The exploration of brain networks, particularly in preterm infants, and contrasting analyses between neonates and adults offer profound insights into the brain's developmental dynamics and structural configuration. This body of research reveals a significant transition in preterm infants' brain activity, shifting from predominantly low‐frequency waves to higher‐frequency waves, particularly within the theta‐alpha range. This transition is pivotal for differentiating brain activity types and lays the groundwork for novel monitoring and neurodevelopmental enhancement strategies for preterm infants. Moreover, leveraging MRI and EEG technologies, the research discerns a uniform power‐law scaling across various brain signal frequencies in both newborns and adults (Fransson et al., 2013), instrumental in identifying intrinsic activity networks and associating these patterns with the newborn brain's sensory and associative area spatial layout. While EEG demonstrates differences in brain activity between posterior (occipital and parietal cortices) and anterior (frontal cortex) regions in newborns with less granularity than MRI, further EEG studies during newborn sleep indicate that high‐activity periods are marked by robust interregional connections, crucial for brain development, a pattern not discernible in MRI scans (Omidvarnia et al., 2014). This suggests that integrating EEG with MRI offers comprehensive insights into early brain network development, enhancing our comprehension of the intricate processes underpinning brain network formation and maturation in preterm infants.

### 4.5 Strengths and limitations

A primary strength of this study is its innovative approach in synthesizing the research trajectory and advancements in neonatal EEG studies using bibliometric analysis. A variety of visualization tools were utilized to depict the bibliometric findings, presenting a detailed overview of the current state, key issues, and prospective directions in neonatal EEG research. However, the study faces certain limitations. Firstly, the literature search was restricted to the WoSCC database, possibly overlooking pertinent studies in other databases. Secondly, by limiting the retrieval to English‐language articles, the scope may not fully capture the global spectrum of research in this field.

## 5 CONCLUSIONS

Neonatal EEG holds significant research value and application potential. The rapidly increasing number of publications indicates that the global scholarly community increasingly recognizes the importance of EEG studies in neonates. The United States and England emerge as leading contributors, with a notable cooperative spirit prevalent across various nations. Significant progress has been made in utilizing amplitude‐integrated EEG (aEEG) for monitoring brain disorders in neonates, such as hypoxic‐ischemic encephalopathy (HIE). The field of automated epilepsy monitoring has advanced to a stage characterized by a low false seizure detection rate. Despite these advancements, a notable challenge persists in the form of inconsistencies in interpreting raw EEG data among researchers, underscoring an area that requires further standardization and refinement.

## AUTHOR CONTRIBUTIONS

Ruijie Zhang, Xinao Lin, Yunlei Bao, and Lifeng Shi searched the database and analyzed the data. Ruijie Zhang and Lu Zhang wrote the main manuscript text and prepared the table. Feng Jiang, Chuyan Wu, and Jimei Wang designed the study, critically reviewed, and made a modification of the manuscript. All authors read and approved the final manuscript.

## FUNDING

No funding was received for conducting this study.

## CONFLICT OF INTEREST STATEMENT

The authors declare that they have no competing interest.

### PEER REVIEW

The peer review history for this article is available at https://publons.com/publon/10.1002/brb3.3483.

## Data Availability

The datasets used and/or analyzed during the current study are available from the corresponding author on reasonable request.
